# Volcanic Soils as Sources of Novel CO-Oxidizing *Paraburkholderia* and *Burkholderia*: *Paraburkholderia hiiakae* sp. nov., *Paraburkholderia metrosideri* sp. nov., *Paraburkholderia paradisi* sp. nov., *Paraburkholderia peleae* sp. nov., and *Burkholderia alpina* sp. nov. a Member of the *Burkholderia cepacia* Complex

**DOI:** 10.3389/fmicb.2017.00207

**Published:** 2017-02-21

**Authors:** Carolyn F. Weber, Gary M. King

**Affiliations:** ^1^Department of Biological Sciences, Louisiana State UniversityBaton Rouge, LA, USA; ^2^College of Health Sciences, Des Moines UniversityDes Moines, IA, USA

**Keywords:** *Burkholderia*, carbon monoxide, *Paraburkholderia*, volcanic soils

## Abstract

Previous studies showed that members of the *Burkholderiales* were important in the succession of aerobic, molybdenum-dependent CO oxidizing-bacteria on volcanic soils. During these studies, four isolates were obtained from Kilauea Volcano (Hawai‘i, USA); one strain was isolated from Pico de Orizaba (Mexico) during a separate study. Based on 16S rRNA gene sequence similarities, the Pico de Orizaba isolate and the isolates from Kilauea Volcano were provisionally assigned to the genera *Burkholderia* and *Paraburkholderia*, respectively. Each of the isolates possessed a form I *coxL* gene that encoded the catalytic subunit of carbon monoxide dehydrogenase (CODH); none of the most closely related type strains possessed *coxL* or oxidized CO. Genome sequences for *Paraburkholderia* type strains facilitated an analysis of 16S rRNA gene sequence similarities and average nucleotide identities (ANI). ANI did not exceed 95% (the recommended cutoff for species differentiation) for any of the pairwise comparisons among 27 reference strains related to the new isolates. However, since the highest 16S rRNA gene sequence similarity among this set of reference strains was 98.93%, DNA-DNA hybridizations (DDH) were performed for two isolates whose 16S rRNA gene sequence similarities with their nearest phylogenetic neighbors were 98.96 and 99.11%. In both cases DDH values were <16%. Based on multiple variables, four of the isolates represent novel species within the *Paraburkholderia*: *Paraburkholderia hiiakae* sp. nov. (type strain I2^T^ = DSM 28029^T^ = LMG 27952^T^); *Paraburkholderia paradisi* sp. nov. (type strain WA^T^ = DSM 28027^T^ = LMG 27949^T^); *Paraburkholderia peleae* sp. nov. (type strain PP52-1^T^ = DSM 28028^T^ = LMG 27950^T^); and *Paraburkholderia metrosideri* sp. nov. (type strain DNBP6-1^T^ = DSM 28030^T^ = LMG 28140^T^). The remaining isolate represents the first CO-oxidizing member of the *Burkholderia cepacia* complex: *Burkholderia alpina* sp. nov. (type strain PO-04-17-38^T^ = DSM 28031^T^ = LMG 28138^T^).

## Introduction

Soils have long been recognized as important biological sinks for carbon monoxide (CO), a critical reactant in the troposphere, yet the microbiology of soil CO oxidizers remains largely unstudied (e.g., Bartholomew and Alexander, 1982; Bender and Conrad, 1994; Conrad, 1996; King, 1999). Early work by Conrad (1988) established rates of atmospheric CO uptake by different soil types along with some of the controls of uptake, but those studies did not identify populations that were active *in situ* or lead to the isolation and characterization of novel CO oxidizers. More recently, molecular ecological approaches have revealed an unexpectedly large diversity of soil aerobic CO oxidizers, including members of the phylum *Actinobacteria* and class *Ktedonobacteria* (phylum *Chloroflexi*), as well as diverse α-, β-, and δ-Proteobacteria and *Euryuarchaeota* (e.g., King, 2003a; Dunfield and King, 2004; King and Weber, 2007; King et al., 2008; Weber and King, 2010a,b; Quiza et al., 2014; King and King, 2014a,b; McDuff et al., 2016).

Recent work with north temperate deciduous forest soils has been particularly interesting, since it has revealed that a group of δ-Proteobacteria related to the myxobacterium, *Haliangeum ochraceum*, plays significant roles in high-affinity atmospheric CO uptake (Quiza et al., 2014). *Haliangeum ochraceum* is intriguing itself, since it harbors the smallest known *cox* gene operon comprised only of the three structural genes for carbon monoxide dehydrogenase (CODH) and one accessory gene. Whether this or aspects of its CODH structure are related to its capacity of atmospheric CO uptake is unknown. Likewise, whether this group accounts for atmospheric CO uptake in other soils is unknown.

Previous work with a rapidly developing forest colonizing volcanic cinders identified members of the β-Proteobacteria, and *Burkholderiales* in particular, as important contributors to the CO-oxidizing community, a community that as a whole was involved with rapid atmospheric CO consumption (King, 2003b; Weber and King, 2010b, 2012). During these studies several novel CO oxidizers were isolated and their ability to consume atmospheric CO was established. Based on a battery of molecular, biochemical, and physiological analyses, we describe here these and a related isolate as new species within the genera *Burkholderia* and *Paraburkholderia* (Yabuuchi et al., 1992; Sawana et al., 2014). We propose the new isolates as potential models for understanding atmospheric CO oxidation by a widely distributed group of terrestrial β-Proteobacteria.

## Materials and methods

### Isolation

*Paraburkholderia* isolates DNBP6-1^T^, I2^T^, PP52-1^T^, and WA^T^ were isolated by Weber and King (2012) from enrichments initiated with forest soil from a 1959 tephra deposit (Pu'u Puai) located on Kilauea Volcano (19° 24′ 22.5″ N × 155° 15′ 18.2″ W); this site has been described previously (King, 2003b; Gomez-Alvarez et al., 2007; King and Weber, 2008). *Burkholderia* isolate PO-04-17-38^T^ was isolated by F.A. Rainey (University of Alaska, Anchorage) and colleagues from enrichments initiated with soil obtained above the tree line at an altitude of about 4357 m on Pico de Orizaba (Mexico) a dormant stratovolcano (see Callegan et al., 2008 for additional details on the site).

Three strains (WA^T^, I2^T^, and PP52-1^T^) were enriched in basal salts media (King, 2003a) with various carbon sources (WA^T^, xylose; I2^T^ pyruvate; PP52-1^T^; mannose). All enrichments were assayed for CO oxidation after adding CO (100 ppm final concentration) to the headspaces of sealed 160-ml serum bottles, and monitoring headspace concentrations at intervals using gas chromatography (King, 1999). Cultures that oxidized CO were used to inoculate solidified versions of the basal salts media used for the original enrichments. Individual colonies were selected from plates, and used to inoculate small volumes of liquid media that were monitored for CO oxidation using gas chromatography as before. Cultures that oxidized CO were purified further by plating, colony selection, and transfer to liquid media; additional CO uptake assays were conducted as necessary. Strain DNBP6-1^T^ was isolated similarly, except that nutrient broth (0.8 g L^−1^) containing penicillin (500 μg ml^−1^) was used for enrichment. Strain PO-04-17-38^T^ was isolated using a medium comprised of 10% R2A (DSMZ medium 830) at 15°C without regard to its capacity for CO oxidation. It was subsequently identified as a CO oxidizer after screening as above. CO uptake capacity for the isolates was assayed following King and King (2014a) with stationary phase liquid cultures (10 ml in 160-ml serum bottles) amended with approximately 200-ppm headspace CO concentrations; cultures were incubated at 30°C with shaking at 200 rpm; cell protein concentrations were determined at the end of the uptake assays using a kit based on bicinchoninic acid (Pierce Protein Research Products; Thermo Scientific).

### Molecular phylogenetic characterization

Phylogenetic characterizations were initiated with genomic DNA obtained from 2-ml cell suspensions that were harvested by centrifugation (10,000 × g, 1 min). DNA in cell pellets was extracted using a MoBio Ultraclean Microbial DNA Extraction Kit (MoBio Laboratories, Carlsbad, CA) following the manufacturer's recommendations with the exception of an added freeze (−80°C)-thaw (65°C) step (3 cycles) prior to bead-beating.

PCR amplification of 16S rRNA genes was performed with primers 27f and 1492r (Lane, 1991). PCR products were visualized using gel electrophoresis (1% agarose) and GelRed stain (Biotium, Inc., Hayward CA). Products of the correct size were purified using a MoBio Ultraclean PCR Cleanup Kit (MoBio Laboratories, Carlsbad, CA). PCR products were sequenced bidirectionally on an ABI model 3130XL at the Louisiana State University Genomics Facility (Baton Rouge, LA). Sequences were assembled and edited using Sequencher v. 4.8 (Gene Codes Corporation, Ann Arbor, MI). Sequences were deposited in Genbank with the following accession numbers: DNBP6-1^T^, JF763856; I2^T^, JF763857.1; PO-04-17-38^T^, JF763852; PP52-1^T^, JF763849; WA^T^, JF763851.

The SINA alignment tool (Pruesse et al., 2007) was used to align isolate 16S rRNA gene sequences with sequences derived from their closest phylogenetic neighbors and related taxa [determined from the EZtaxon application (Kim et al., 2012)]. Alignments were adjusted manually as necessary using MEGA7 (Tamura et al., 2013). Maximum likelihood analyses were also performed using MEGA7 with a general time reversible model and 100 and 1,000 bootstrap replicates, respectively.

EZTaxon (Kim et al., 2012) was used to obtain 16S rRNA gene sequences similarities for a set of type strains representing the phylogenetic neighborhood of the CO-oxidizing isolates. Genomes for strains with 16S rRNA gene sequence similarities > 98.0% were then used to generate parallel pairwise comparisons of average nucleotide identity (ANI). ANI was calculated using the comparative genome toolkit from the Integrated Microbial Genomes/Microbiome Samples website (https://img.jgi.doe.gov/cgi-bin/m/main.cgi).

*RecA* genes were amplified using primers Bur3 (forward) and Bur4 (reverse) to further clarify the phylogenetic positions of isolates DNBP6-1^T^, I2^T^, PP52-1^T^, and WA^T^; the PCR protocol followed the methods of Payne et al. (2005). Purified amplicons of the correct size (385 bp) were sequenced bi-directionally as above. MUSCLE was used in the MEGA7 platform (Tamura et al., 2013) to align partial *recA* gene sequences for the isolates' close phylogenetic neighbors. Phylogenetic analyses were also performed using MEGA7. Sequences were deposited in Genbank with the following accession numbers: DNBP6-1^T^, KY305132; I2^T^, KY305131; PP52-1^T^, KY305133; WA^T^, KY3051130.

### Morphological and physiological characterization

Routine microscopy and staining methods were used for basic isolate characterization (Gerhardt et al., 1994). pH ranges suitable for growth were determined by cultivating the isolates in R2A media with pH adjusted between values of 5.5 and 9.5. A phosphate buffer (0.1 M) was used to prepare media with pH values from 5.5 to 6.5; a CO_2_/sodium bicarbonate/sodium carbonate buffer was used to prepare media with pH values from 7.5 to 9.5. Temperature optima were assessed similarly using cultures grown from 5 to 50°C (R2A medium at pH 6.5).

Sole carbon source metabolism patterns were assessed with Biolog GN2 plates (Biolog, Inc.; Hayward CA, USA) following the manufacturer's recommendations. Sole carbon source assimilation, enzymatic reactions (including oxidase and catalase), nitrate reduction, and other biochemical traits were also assayed with API 20NE strips following the manufacturer's recommendations (bioMérieux SA; Marcy l'Etoile, France). In addition, the ability of isolates to grow with selected sole carbon and energy sources was assessed in liquid culture with the basal salts medium above containing 25 mM of individual carbon sources.

### Phospholipid fatty acid characterization

Phospholipid fatty acid analyses for the isolates were carried out by the DSMZ Identification Service using standard extraction and analytical methods (Miller, 1982; Kuykendall et al., 1988). After methylation and gas chromatographic quantitation, individual fatty acids were identified using the standard protocol of the Sherlock Microbial Identification System (MIDI Inc.).

### DNA G+C content and DNA-DNA hybridization

DNA base composition (mol% G+C) for all strains was also determined by the Identification Service of the DSMZ (Braunschweig, Germany) using the method of Mesbah et al. (1989). The Identification Service of DSMZ performed DNA-DNA hybridizations for two isolates, *Paraburkholderia* sp. DNBP6-1^T^ and *Paraburkholderia* sp. PP52- 1^T^, with their closest phylogenetic neighbors (*P. bryophila* LMG 23644^T^ and *P. mimosarum* DSM 21841^T^, respectively) using the protocols of Cashion et al. (1977), De Ley et al. (1970) and Huss et al. (1983).

## Results and discussion

**Figure 1:**
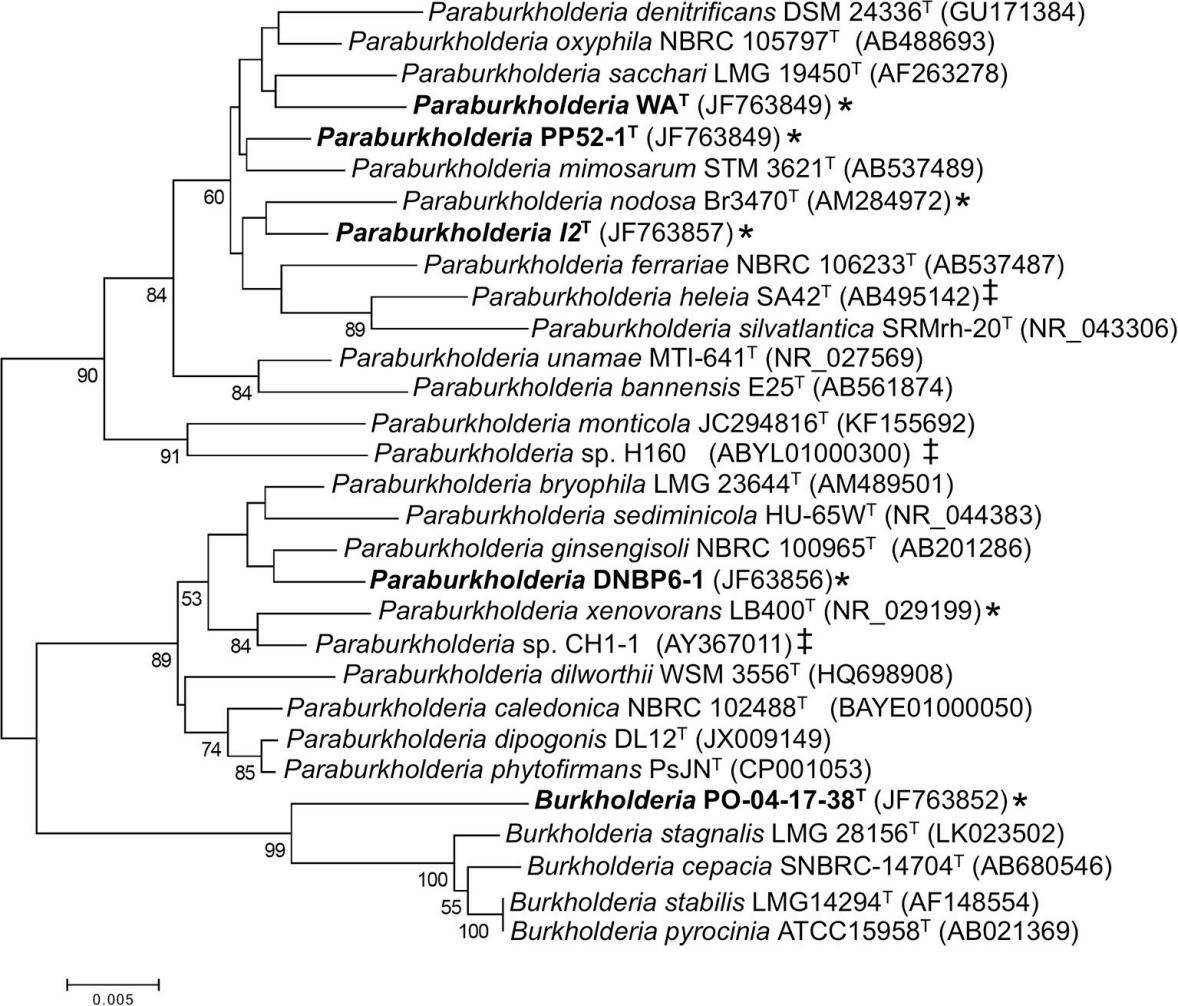
**Maximum likelihood analysis of partial 16S rRNA gene sequences from CO-oxidizing ***Paraburkholderia*** and ***Burkholderia*** isolates, and related taxa**. Bootstrap support ≥ 50% is indicated below the branches. A discrete Gamma distribution was used to model evolutionary rate differences among sites. All gapped positions and positions with missing or ambiguous data were removed, leaving 1263 positions in the final dataset. Asterisks (^*^) indicate confirmed CO oxidizers; ‡ indicates isolates with form I CO dehydrogenase genes identified in their genome sequences.

Phylogenetic analyses of 16S rRNA gene sequences (Figure 1) showed that the Pico de Orizaba isolate (PO-04-17-38^T^) clustered with the genus *Burkholderia*. Results from analyses conducted with EZtaxon (Kim et al., 2012) further showed that PO-04-17-38^T^ was most closely related to *B*. *stabilis* LMG 14294^T^ with a 16S rRNA gene sequence similarity of 97.49%. This level of similarity is considered consistent with species novelty and is less than similarity values (i.e., > 98.7–99.0%) for which DNA-DNA hybridization assays have been proposed for establishing species distinctions (Stackebrandt and Ebers, 2006).

Phylogenetic analyses also showed that the Kilauea Volcano isolates clustered with the genus *Paraburkholderia*. Pairwise comparisons revealed that each of the isolates shared 16S rRNA gene sequence similarities no greater than 98.1% with other isolates, a difference consistent with species demarcation (Stackebrandt and Ebers, 2006). However, 16S rRNA gene sequence similarities for the isolates and their closest type species phylogenetic neighbors were somewhat higher: 98.53% for *Paraburkholderia* sp. I2^T^ vs. *P. oxyphila* NBRC 105797^T^; 98.80% for *Paraburkholderia* sp. WA^T^ vs. *P. oxyphila* NBRC 105797^T^; 98.96% for *Paraburkholderia* sp. PP52-1^T^ vs. *P. mimosarum* DSM 21841^T^; and 99.11% for *Paraburkholderia* sp. DNBP6-1^T^ vs. *P. bryophila* LMG 223644^T^.

To determine whether these similarities were consistent with the delineation of novel *Paraburkholderia* species, 16S rRNA gene sequence similarities were generated using EZTaxon (Kim et al., 2012) for pairs of type species in the phylogenetic neighborhood of the isolates. The maximum sequence similarity in this set of comparisons was 98.93%. For all pairs with similarities ≥ 98.0–98.93%, genome sequences were used to generate a set of average nucleotide identities (ANI; Supplementary Table 1). ANI did not exceed 95% (a recommended lower cutoff for species differentiation; Richter and Rosselló-Móra, 2009; Kim et al., 2014; Yarza et al., 2014) in any comparison (Supplementary Table 1, Supplementary Figure 1). Moreover, a more extensive analysis of ANI and 16S similarities involving multiple genera from multiple phyla yielded comparable resuts (Kim et al., 2014). This observation supports designation of isolates I2^T^ and WA^T^ as novel species based on similarities with their closest phylogenetic neighbor, *P*. *oxyphila* NBRC 105797^T^ (98.53 and 98.80%, respectively).

However, similarities for isolates PP52-1^T^ and DNBP6-1^T^ and their closest neighbors exceed 98.3% (see above). DNA-DNA hybridizations (DDH) performed for these two isolates and their nearest neighbors yielded values of 15.8% (*Paraburkholderia* sp. PP52-1^T^ vs. *P. mimosarum* DSM 21841^T^) and 12.4% (*Paraburkholderia* sp. DNBP6-1^T^ vs. *P. bryophila* LMG 223644^T^); these values are consistent with species level demarcation (e.g., Stackebrandt and Ebers, 2006).

**Figure 2:**
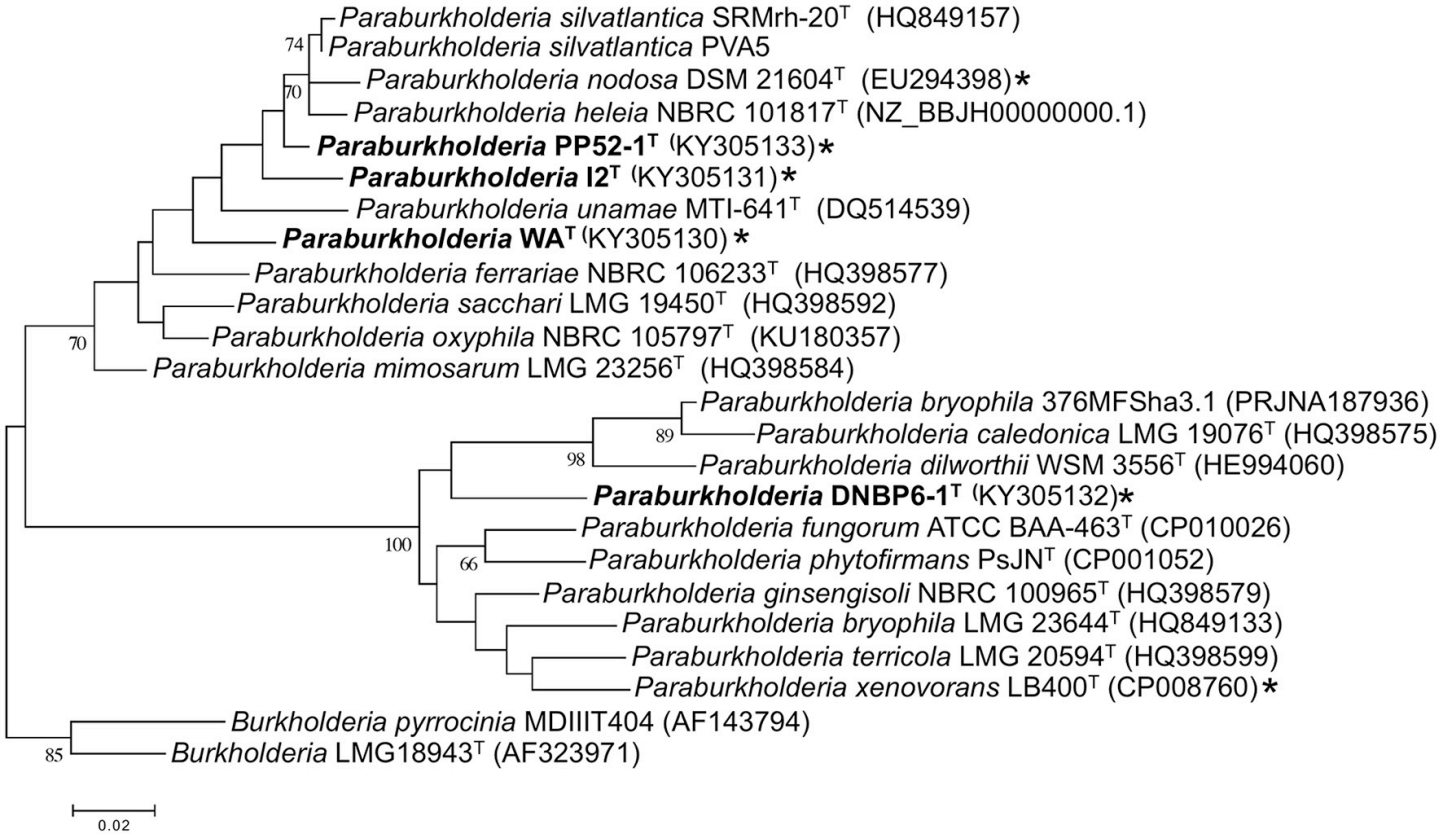
**Maximum likelihood analysis of partial ***recA*** gene sequences from CO-oxidizing ***Paraburkholderia*** and ***Burkholderia*** isolates, and related taxa**. Bootstrap support is shown below the branches for values ≥ 50%. All gapped positions and missing data were eliminated resulting in 331 positions for the analysis. ^*^indicates CO oxidier.

Phylogenetic analyses of partial *recA* gene sequences were also consistent with species level differentiation (Figure 2). Primers Bur3 and Bur4 have been previously shown to discriminate successfully among numerous *Burkholderia* (*Paraburkholderia*) species (Payne et al., 2005; Hall et al., 2015). Although the topologies of phylogenetic trees in this study and that of Payne et al. (2005) did not have strong bootstrap support, the overall topologies were consistent with the topologies of phylogenetic trees based on 16S rRNA gene sequences, and the isolates in this study were clearly distinct from related taxa (Figure 2).

BLAST analyses of *Burkholderia* and *Paraburkholderia* genomes and previously published culture-based assays of CO oxidation potential (Weber and King, 2012) showed that none of the closest type strain phylogenetic neighbors of the isolates in this study harbored form I carbon monoxide dehydrogenase (*cox*) genes or oxidized CO. However, genome analyses revealed form I *cox* genes in *P*. *heleia* SA42^T^, *P*. *nodosa* LMG 23741^T^, *Paraburkholderia* sp. CH1-1, and *Paraburkholderia* sp. H160. Previous studies have confirmed CO oxidation by *P*. *nodosa* LMG 23741^T^, *P*. *xenovorans* LB400^T^, and *Paraburkholderia* sp. LUP (King, 2003a; Weber and King, 2012). All of these isolates are phylogenetically distinct from those in this study (Figure 1).

CO uptake capacity varied substantially among the isolates (nmol CO mg protein^−1^ h^−1^; mean ± standard error): PO-04-17-38^T^, 20.4 ± 0.5 WA^T^, 27.7 ± 5.0; I2^T^, 88.8 ± 37.5; PP52-1^T^, 155.2 ± 24.2; DNBP6-1^T^, 226.5 ± 85.2. However, these values fall within previously reported ranges for other CO-oxidizing isolates (Weber and King, 2007; King and King, 2014a,b). Although rates for PO-04-17-38^T^ and WA^T^ were relatively low compared to the other isolates, the physiological and ecological significance of these differences are unknown at present.

All isolates were Gram-negative, non-spore forming, non-motile, CO-oxidizing rods [CO oxidation capacity was reported previously by Weber and King (2012)]. All were catalase positive, and all but PP52-1^T^ were oxidase positive (Table 1). Colonies formed on solid pyruvate-yeast extract media [PYE, (Weber and King, 2012)] were circular with entire margins, with white coloration for I2^T^, DNBP6-1^T^, and PP52-1^T^; colonies for WA^T^ and PO-04-17-38^T^ were off-white.

**Table 1:** **Phenotypic characteristics of CO-oxidizing ***Paraburkholderia*** and ***Burkholderia*** isolates and phylogenetic neighbors**.

**Trait**	**1**	**2**	**3**	**4**	**5**	**6**	**7**	**8**	**9**	**10**	**11**
Motility	−	−	−	−	−	−	−	nr	−	−	−
Oxidase reaction	−	+	+	+	+	−	+	+	+	+	+
Nitrate reduction	+	+	−	+	+	−	−	v	−	+	+
G+C content (mol%)	63.7	63.1	60.3	64.9	64.9	64	62	64.8	68−69	66.8	67
Temp optimum (°C)	30	30	30	30	25	30	nr	nr	20−24	nr	nr
Range	15− <40	15− <40	15−40	15−45	5− <45	15−40	nr	nr	6−32	nr	nr
pH optimum	6.5	5	7.5	6.5	6.5	nr	nr	nr	4.7−5.2	nr	nr
Range	5.7− <8.5	5.7−8.0	5.7−8.5	5.7−8.5	5.7−8.0	3.5−8.5	nr	nr	3.2−6.6	nr	nr
Enzyme reactions:											
Arginine dihydrolase	−	−	±	−	−	−	−	−	−	−	−
Gelatinase	−	−	−	+	−	−	−	−	−	+	+
Esculin hydrolysis	−	−	−	+	−	+	−	−	−	−	−
β-galactosidase	−	−	+	−	+	−	+	−	−	−	+
Assimilation:											
Glucose	+	+	−	+	−	+	+	+	+	+	+
Arabinose	+	+	−	−	−	−	+	+	−	+	+
Mannose	+	+	−	+	−	+	+	v	+	+	+
Mannitol	+	+	+	+	−	+	+	+	+	+	+
N-acetylglucosamine	+	+	+	+	−	+	+	+	−	+	+
Malate	−	−	−	+	−	−	+	+	+	+	+
Gluconate	−	+	−	+	−	+	+	v	−	+	+
Caprate	+	+	−	+	−	+	+	−	+	+	+
Adipate	+	−	−	±	−	+	v	−	+	+	+
Maltose	±	+	−	+	−	+	−	−	+	−	−
Citrate	−	−	−	±	−	+	+	−	+	+	+
Phenylacetate	+	−	−	+	−	+	−	v	+	+	+

*1 = PP52-1^T^, 2 = I2^T^, 3 = DNBP6-1^T^, 4 = WA^T^, 5 = PO-04-17-38^T^, 6 = P. oxyphila OX-01^T^ (Otsuka et al., 2011); 7 = P. bryophila LMG 23644^T^ (Vandamme et al., 2007a); 8 = P. mimosarum LMG 23256^T^ (Chen et al., 2006); 9 = B. stabilis LMG 14294^T^ (Henry et al., 2001); 10 = B. ambifaria LMG19812^T^ (Coenye et al., 2001); 11 = B. diffusa LMG24065^T^ (Vanlaere et al., 2008a). All isolates were catalase positive; nr = not reported; v = reported as variable among strains, type strain response not indicated; ± = variable for multiple trials*.

Three isolates (PO-04-17-38^T^, PP52-1^T^, and WA^T^) grew with pH optima at 6.5, while the optimum for the remaining two was 7.5; all grew at pH 5.7 and showed an upper limit of pH 8–pH 8.5 (Table 1). Isolate PO-04-17-38^T^ grew with a notably lower temperature optimum (25°C) and minimum (5°C) than for other isolates in this study, but its response to temperature was similar to that of its closest phylogenetic neighbor, *Burkholderia stabilis* LMG 14294^T^ (Table 1). The remaining isolates grew optimally at 30°C with a minimum at 15°C and an upper limit of 40°–45°C; similar values were observed for their phylogenetic neighbors (Table 1).

Positive reactions on Biolog GN-2 plates varied from a low of 20 substrates for PO-04-17-38^T^ (mostly sugars and a few simple organic acids) to 72 of 95 substrates for DNBP6-1^T^ (Supplementary Table 2). With the exception of PO-04-17-38^T^, the isolates used a variety of sugars, sugar derivatives, organic acids, and amino acids that reflected the broad substrate utilization patterns reported for *Paraburkholderia* (e.g., Chen et al., 2007; Vandamme et al., 2007b; Compant et al., 2008; Vanlaere et al., 2008b; Aizawa et al., 2011; Otsuka et al., 2011). Nonetheless, substrate use differed for each of the isolates, and for three of the isolates when compared with their phylogenetic neighbors. Previously published BIOLOG GN-2 substrate utilization reactions facilitated comparisons between strains PP52-1^T^ and *P. mimosarum* DSM 21841^T^, I2^T^ and *P. nodosa* LMG 23741^T^, and DNBP6-1^T^ and *P*. *bryophila* LMG 23644^T^ (Supplementary Table 2). At least 10% of the 95 substrate reactions differed in each of these paired comparisons. It must be noted however that strain variability and variability among assays could reduce or increase these differences.

Results from API 20NE strips also revealed differences in substrate assimilation among the CO-oxidizing isolates and some of their close phylogenetic neighbors. Notably, isolate PO-04-17-38^T^ did not assimilate any of the substrates in the panel, while its nearest phylogenetic neighbor, *B*. *stabilis* LMG 14294^T^, assimilated all but mannitol, N-acetylglucosamine and gluconate (Table 1). The lack of substrate assimilation by PO-04-17-38^T^ contrasts with its ability to oxidize substrates in Biolog GN2 plates (Supplementary Table 2), and to grow in liquid culture with arabinose, glucose, mannitol, and mannose (Supplementary Table 3). The lack of substrate assimilation by PO-04-17-38^T^ in the API 20NE panel was repeatable, however, which suggests that assay conditions for the API tests do not reliably reflect the capacity of PO-04-17-38^T^ to use substrates.

Strain I2^T^ differed from its closest phylogenetic neighbor, *P*. *oxyphila* OX-01^T^, in its ability to assimilate arabinose, but not adipate, citrate or phenylacetate (Table 1). Strain PP52-1^T^ differed from its closest phylogenetic neighbor, *P. mimosarum* DSM 21841^T^, in its ability to assimilate mannose, caprate, adipate, and phenylacetate, but not malate (Table 1). Strain DNBP6-1^T^ differed from its closest phylogenetic neighbor, *P. bryophila* LMG 23644^T^, in its inability to assimilate glucose, arabinose, mannose, malate, gluconate, caprate, or citrate (Table 1). Strain WA^T^ differed from its closest phylogenetic neighbor, *P*. *oxyphila* OX-01^T^, in its inability to assimilate malate (Table 1).

The ability of the isolates to grow on various substrates in liquid culture (Supplementary Table 3) largely paralleled observations from Biolog GN2 plates (Supplementary Table 2). Strain PO-041783^T^ grew with the fewest substrates (11 of 39, mostly sugars and a few organic acids) and was inhibited by several, while the *Paraburkholderia* isolates grew with 19–22 of 39 substrates. None of the strains were able to grow with glycine, phthalate, or solvents and alcohols, but two strains (I2^T^ and WA^T^) were able to use dimethylamine and trimethylamine.

Clear distinctions were observed for the fatty acid compositions of each of the CO-oxidizing strains relative to compositions reported for each of their closest phylogenetic neighbors (Table 2). Strain PP52-1^T^ contained greater amounts of C_16:0_, C_17:0cyclo_, and C_16:1−2OH_, and lesser amounts of C_18:1ω7c_ fatty acids than *P. mimosarum* DSM21841^T^; PP52-1^T^ also lacked C_16:0−2OH_. Strain I2^T^ contained modest levels of C_16:1−2OH_, which was absent from *P*. *oxyphila* OX-01^T^, and considerably less C_18:1ω7c_. Strain WA^T^ also contained C_18:1−2OH_ and modest levels of C_16:1−2OH_, which were lacking in *P*. *oxyphila* OX-01^T^ lipids. Strain DNBP6-1^T^ contained notably higher amounts of C_17:0cyclo_ and C_19:0cycloω8c_, and lower amounts of C_18:1ω7c_ and isoC_15:0−2OH_/C_16:1ω7c_ (sum feature 3) than *P*. *bryophila* LMG 23644^T^; strain DNBP6-1^T^ also lacked C_16:0−2*OH*_. Strain PO-141738^T^ contained sum feature 3, C_18:1ω7c_ and C_16:1−2OH_, which were absent in *B*. *stabilis* LMG 14294^T^, but it lacked C_16:0−2OH_, C_16:1ω7c_, C_18:1_, and C_18:1−2OH_.

**Table 2:** **Fatty acid composition for novel ***Paraburkholderia*** and ***Burkholderia*** isolates and related strains**.

**Fatty acid**	**1**	**2**	**3**	**4**	**5**	**6**	**7**	**8**	**9**	**10**
C_14:0_	4.8	3.6	5.2	4.00	3.7	1.0	4.7	1.2	4.4	3.4
C_14:0−3OH_	4.5	5.2	4.7	4.80	4.7	4.7	5.3	6.3	5.4	7.2
C_16:0_	29.1	19.1	23.5	18.3	20.9	22.6	18.3	24.2	25.6	15.0
C_16:0−2OH_	–	1.6	–	–	–	1.5	–	–	4.5	–
C_16:0−3OH_	3.1	4.2	3.6	4.00	3.7	3.9	3.7	4.3	5.7	5.9
C_17:0_	0.1	–	–	–	0.1	–	0.1	–	–	–
C_17:0cyclo_	14.0	3.3	21.9	18.40	27.7	11.0	16.2	13.7	22.3	5.1
C_18:0_	0.2	–	0.2	–	0.3	–	0.2	0.6	–	1.6
C_19:0iso_	0.2	–	0.4	–	0.5	–	0.1	0.6	–	–
C_19:0cycloω8c_	2.0	1.8	10.8	2.70	22.2	2.7	6.9	9.8	15.3	1.7
C_16:1ω5c_	–	–	0.6	–	0.6	–	0.6	–	–	–
C_16:1ω7c_	–	–	–	–	–	–	–	–	3.3	22.5
C_16:1−2OH_	5.4	0.9	5.3	–	3.0	2.1	4.6	5.2	–	1.5
anteisoC_17:1ω9c_	0.1	–	0.1	–	–	–	0.1	–	–	–
C_18:1_	–	–	–	–	–	–	–	–	10.6	–
C_18:1ω5c_	0.1	–	0.2	–	0.1	–	0.2	–	–	–
C_18:1ω7c_	21.8	45.6	17.8	34.60	6.9	22.6	31.8	17.7	–	32.1
C_18:1−2OH_	0.8	1.0	–	–	0.6	–	1.1	–	2.4	2.4
C_18:1−11methylω7c_	–	–	0.2	–	0.6	–	0.1	0.5	–	–
C_20:2ω6.9c_	–	–	0.2	–	0.4	–	–	0.7	–	–
C_16:1ω7c_/isoC_15:0−2OH_	13.1	12.6	3.7	6.40	2.7	22.2	5.8	9.8	–	–
Total	99.3	98.9	99.6	93.10	98.7	94.0	99.8	94.3	99.5	97.9

*Strain designations as follows: 1, PP52-1^T^; 2, P. mimosarum LMG 23256^T^ (Chen et al., 2006); 3, I2^T^; 4, P. oxyphila OX-01^T^ (Otsuka et al., 2011); 5, DNBP6-1^T^; 6, P. bryophila LMG 23644^T^ (Lu et al., 2012); 7, WA^T^; 8, PO-04-17-38^T^; 9, B. stabilis LMG 14294^T^(Henry et al., 2001); 10, B. ambifaria LMG19812^T^ (Coenye et al., 2001)*.

G+C contents varied between 60.3 and 64.9 mol% for the various CO-oxidizing isolates. These values were consistent with results reported for other *Paraburkholderia* and *Burkholderia* (Table 1).

The isolates obtained in this study share multiple characteristics with members of the genera *Burkholderia* and *Paraburkholderia* into which they were placed on the basis of 16S rRNA gene analyses (Figure 1). Although several CO-oxidizing members of *Paraburkholderia* have been identified previously (King, 2003a), PO-04-17-38^T^ represents the first CO-oxidizing member of the *Burkholderia*, and in particular the *Burkholderia cepacia* complex, a group that harbors a number of important pathogens (Peeters et al., 2016). This observation is notable, since genomic sequencing of a large number of *Burkholderia* has yet to reveal any putative CO oxidizers, while at least 5 putative CO oxidizers have been identified among the *Paraburkholderia*. In addition, a phylogenetic analysis has shown that the PO-04-17-38^T^ *coxL* gene clusters most closely with *coxL* from isolate DBNP6-1^T^, which suggests that a horizontal gene transfer event from *Paraburkholderia* to PO-04-17-38^T^ might account for its apparently unusual capacity to oxidize CO.

The collective phenotypic, physiological, phylogenetic and biochemical results indicate that the CO-oxidizing *Burkholderia* and *Paraburkholderia* strains isolated during this study represent novel species, for which the following designations are proposed: *Burkholderia alpina* sp. nov. (the type strain is PO-04-17-38^T^ = DSM 28031^T^ = LMG 28138^T^); *Paraburkholderia hiiakae* sp. nov. (the type strain is I2^T^ = DSM 28029^T^ = LMG 27952^T^); *Paraburkholderia paradisi* sp. nov. (the type strain is WA^T^ = DSM 28027^T^ = LMG 27949^T^); *Paraburkholderia metrosideri* sp. nov. (the type strain is DNBP6-1^T^ = DSM 28030^T^ = LMG 28140^T^); *Paraburkholderia peleae* sp. nov. (the type strain is PP52-1^T^ = DSM 28028^T^ = LMG 27950^T^).

### Description of *Burkholderia alpina* sp. nov.

*Burkholderia alpina* (al.pi'na. L. fem. adj. alpina, pertaining to the Alps and generally from or inhabiting mountainous regions, especially above the tree line, alpina referring to an isolate from an alpine altitude).

Cells are Gram-negative, non-sporing, non-motile rods, catalase, and oxidase positive. Colonies are circular, entire, off-white. The following carbon sources supported growth at 25 mM in a basal salts medium: alanine, arabinose, galactose, glucose, glutamate, β-hydroxybutyrate, lactate, mannitol, mannose, pyruvate, ribose, and tartrate. The following carbon sources did not support growth: acetate, aspartate, benzoate, betaine, citrate, dimethylamine, formate, gluconate, glucuronate, glycine, α-keto-glutarate, isopropanol, lactose, malonate, maltose, methanol, methylamine, phenylalanine, phthalate, propionate, succinate, trimethylamine, and valine. Weak growth was observed with fructose, glycerol, malate, and proline. Temperature optimum 25°C with growth at 5°C and at 35°C but not 45^*o*^C. pH optimum 6.5 with growth at pH 5.7 but not pH 8.5. Carbon monoxide is oxidized aerobically. The major cellular fatty acids (≥1% of the total) include: C_14:0_, C_14:0−3OH_, C_16:0_, C_17:0cyclo_, C_16:1−2OH_, C_16:0−3OH_, C_18:1−ω7c_, C_19:0−cycloω8c_, and iso-C_15:0−2OH_/C_16:1−ω7c_. The DNA G+C content of the type strain is 64.9 mol%. The type strain, PO-04-17-38^T^ (= DSM 28031^T^ = LMG 28138^T^), was isolated from volcanic soils from Pico de Orizaba (Mexico). The GenBank/EMBL/DDBJ accession number for the 16S rRNA gene sequence of strain PO-04-17-38^T^ is JF763852.1.

### Description of *Paraburkholderia hiiakae* sp. nov.

*Burkholderia hiiakae* (hi.i.a'kae. Hawaiian N.L. fem. gen. n. from Hiiakaikapoliopele, a Hawaiian goddess of hula dancers, chants and sorcery, hiiakae, honoring the Hawaiian goddess of hula dancers).

Cells are Gram-negative, non-sporing, non-motile rods, catalase, and oxidase positive. Colonies are circular, entire, white. The following carbon sources supported growth at 25 mM in a basal salts medium: alanine, arabinose, aspartate, benzoate, citrate, formate, gluconate, glucose, α-keto-glutarate, glycerol, glutamate, β-hydroxybutyrate, lactate, malate, mannitol, phenylalanine, proline, propionate, ribose, succinate, and tartrate. The following carbon sources did not support growth: arabinose, aspartate, betaine, glucuronate, glutamate, glycine, isopropanol, lactose, maltose, methanol, methylamine, phthalate, and valine. Weak growth was observed with dimethylamine, fructose, galactose, malonate, mannose, and trimethylamine. Temperature optimum 30°C with growth at 15°C and no growth at 40°C. pH optimum 7.5 with growth at pH 5.7 but not pH 8.5. Carbon monoxide is oxidized aerobically. The major cellular fatty acids (≥1% of the total) include: C_14:0_, C_14:0−3OH_, C_16:0_, C_17:0cyclo_, C_16:1−2OH_, C_16:0−3OH_, C_18:1−ω7c_, C_19:0−cycloω8c_, C_18:1−2OH_, and iso-C_15:0−2OH_/C_16:1−ω7c_. The DNA G+C content of the type strain is 63.1 mol%. The type strain, I2^T^ (= DSM 28029^T^ = LMG 27952^T^), was isolated from volcanic soils from Kilauea Volcano (Hawai‘i, USA). The GenBank/EMBL/DDBJ accession number for the 16S rRNA gene sequence of strain I2^T^ is JF763857.1.

### Description of *Paraburkholderia metrosideri* sp. nov.

*Paraburkholderia metrosideri* (me.tro.si.de'ri. N.L. fem. gen. n. metrosideri of *Metrosideros*, the genus *Metrosideros polymorpha*, the ohia tree).

Cells are Gram-negative, non-sporing, non-motile rods, catalase, and oxidase positive. Colonies are circular, entire, white. The following carbon sources supported growth at 25 mM in a basal salts medium: alanine, arabinose, benzoate, betaine, fructose, galactose, gluconate, glucuronate, glucose, α-keto-glutarate, glycerol, β-hydroxybutyrate, lactate, lactose, malate, mannitol, mannose, proline, propionate, pyruvate, ribose, succinate, and tartrate. The following carbon sources did not support growth: aspartate, citrate, dimethylamine, formate, glycine, isopropanol, methanol, methylamine, phenylalanine, phthalate, trimethylamine, and valine. Weak growth was observed with acetate, glutamate, malonate, and maltose. Temperature optimum 30°C with growth at 15°C and at 40°C but not 45°C. pH optimum 7.5 with growth at pH 5.7 and pH 8.5 but not 9.5. Carbon monoxide is oxidized aerobically. The major cellular fatty acids (≥1% of the total) include: C_14:0_, C_14:0−3OH_, C_16:0_, C_17:0cyclo_, C_16:1−2OH_, C_16:0−3OH_, C_18:1−ω7c_, C_19:0−cycloω8c_, and iso-C_15:0−2OH_/C_16:1−ω7c_. The DNA G+C content of the type strain is 60.3 mol%. The type strain, DNBP6-1^T^ (= DSM 28030^T^ = LMG 28140^T^), was isolated from volcanic soils from Kilauea Volcano (Hawai‘i, USA). The GenBank/EMBL/DDBJ accession number for the 16S rRNA gene sequence of strain DNBP6-1^T^ is JF763856.1.

### Description of *Paraburkholderia paradisi* sp. nov.

*Paraburkholderia paradisi* (L. masc. gen. n. pa.ra.di'si of paradise).

Cells are Gram-negative, non-sporing, non-motile rods, catalase, and oxidase positive. Colonies are circular, entire, off-white. The following carbon sources supported growth at 25 mM in a basal salts medium: alanine, benzoate, citrate, dimethylamine, fructose, galactose, gluconate, glucose, glutamate, α-keto-glutarate, glycerol, glutamate, β-hydroxybutyrate, lactate, mannitol, mannose, proline, pyruvate, succinate, trimethylamine, and valine. The following carbon sources did not support growth: arabinose, aspartate, betaine, glycine, isopropanol, lactose, maltose, methanol, methylamine, phenylalanine, phthalate, and ribose. Weak growth was observed with acetate, formate, glucuronate, malate, malonate, propionate, and tartrate. Temperature optimum 30°C with growth at 15°C and at growth at 45°C but not 50°C. pH optimum 6.5 with growth at pH 5.7 and pH 8.5 but not 9.5. Carbon monoxide is oxidized aerobically. The major cellular fatty acids (≥1% of the total) include: C_14:0_, C_14:0−3OH_, C_16:0_, C_17:0cyclo_, C_16:1−2OH_, C_16:0−3OH_, C_18:1−ω7c_, C_19:0−cycloω8c_, C_18:1−2OH_, and iso-C_15:0−2OH_/C_16:1−ω7c_. The DNA G+C content of the type strain is 64.9 mol%. The type strain, WA^T^ (= DSM 28027^T^ = LMG 27949^T^), was isolated from volcanic soils from Kilauea Volcano (Hawai‘i, USA). The GenBank/EMBL/DDBJ accession number for the 16S rRNA gene sequence of strain WA^T^ is JF763851.1.

### Description of *Paraburkholderia peleae* sp. nov.

*Paraburkholderia peleae* (pe.le'ae. Hawaiian N.L. gen. n. from Pelehonuamea, Pele of the sacred land, Hawaiian goddess of volcanoes, peleae honoring the goddess Pele).

Cells are Gram-negative, non-sporing, non-motile rods, catalase positive, and oxidase negative. Colonies are circular, entire, white. The following carbon sources supported growth at 25 mM in a basal salts medium: acetate, alanine, arabinose, aspartate, betaine, fructose, galactose, gluconate, glucose, α-keto-glutarate, glycerol, β-hydroxybutyrate, lactate, malate, malonate, mannitol, mannose, proline, propionate, pyruvate, ribose, succinate, and tartrate. The following carbon sources did not support growth: benzoate, citrate, dimethylamine, formate, glucuronate, glutamate, glycine, isopropanol, lactose, maltose, methanol, methylamine, phenylalanine, phthalate, trimethylamine, and valine. Temperature optimum 30°C with growth at 15°C and no growth at 40°C. pH optimum 6.5 with growth at pH 5.7 but not pH 8.5. Carbon monoxide is oxidized aerobically. The major cellular fatty acids (≥1% of the total) include: C_14:0_, C_14:0−3OH_, C_16:0_, C_17:0cyclo_, C_16:1−2OH_, C_16:0−3OH_, C_18:1−ω7c_, C_19:0−cycloω8c_, and iso-C_15:0−2OH_/C_16:1−ω7c_. The DNA G+C content of the type strain is 63.7 mol%. The type strain, PP52-1^T^ (= DSM 28028^T^ = LMG 27950^T^), was isolated from volcanic soils from Kilauea Volcano (Hawai‘i, USA). The GenBank/EMBL/DDBJ accession number for the 16S rRNA gene sequence of strain PP52-1^T^ is JF763849.1.

## Author contributions

CW collected samples, enriched isolates, characterized isolates, analyzed data, and wrote the manuscript. GK collected samples, enriched isolates, characterized isolates, analyzed data, and wrote the manuscript.

### Conflict of interest statement

The authors declare that the research was conducted in the absence of any commercial or financial relationships that could be construed as a potential conflict of interest. The reviewer DS and handling Editor declared their shared affiliation, and the handling Editor states that the process nevertheless met the standards of a fair and objective review.
