# Supplementary material for: Volcanic Soils as Sources of Novel CO-Oxidizing Paraburkholderia and Burkholderia: Paraburkholderia hiiakae sp. nov., Paraburkholderia metrosideri sp. nov., Paraburkholderia paradisi sp. nov., Paraburkholderia peleae sp. nov., and Burkholderia alpina sp. nov. a Member of the Burkholderia cepacia Complex
Source: Front Microbiol. 2017 Feb 21;8:207. doi: 10.3389/fmicb.2017.00207 (PMC5318905; doi:10.3389/fmicb.2017.00207)
Supplement: Supplementary file 1 [file Table1.DOCX]

Supplementary Table 1. 16S rRNA gene similarities (%) and average nucleotide indices (ANI, %) for comparisons between various *Paraburkholderia* type species. 16S rRNA gene similarities were determined using EZTaxon (Kim et al., 2012); ANI was determined using the IMG toolkit.

| **Species 1** | **Species 2** | **16S rRNA** | **ANI** |
| --- | --- | --- | --- |
| *P. ginsengisoli* NBRC 100965^T^ | *P. caledonica* NBRC102488^T^ | 98.06 | 85.29 |
| *P. dilworthii* WSM3556^T^ | *P. caledonica* NBRC102488^T^ | 98.72 | 89.91 |
|  | *P*. *fungorum* NBRC102489^T^ | 98.11 | 84.43 |
|  | *P. phytofirmans* PsJN^T^ | 98.11 | 85.48 |
| *P. caledonica* NBRC102488^T^ | *P. xenovorans* LB400^T^ | 98.19 | 85.03 |
|  | *P*. *fungorum* NBRC102489^T^ | 98.39 | 83.90 |
|  | *P. phytofirmans* PsJN^T^ | 98.66 | 84.94 |
| *P. xenovorans* LB400^T^ | *P*. *fungorum* NBRC102489^T^ | 98.25 | 86.18 |
|  | *P. phytofirmans* PsJN^T^ | 98.86 | 89.70 |
|  | *P*. *terricola* LMG20954^T^ | 98.25 | 86.18 |
| *P*. *fungorum* NBRC102489^T^ | *P. phytofirmans* PsJN^T^ | 98.22 | 86.48 |
|  | *P*. *terricola* LMG20954 | 98.09 | 86.83 |
|  | *P*. *phenoliruptrix* BR3459a^T^ | 98.23 | 84.45 |
|  | *P*. *megapolitana* LMG23650^T^ | 98.57 | 81.03 |
|  | *P*. *phenazinium* LMG2247^T^ | 98.54 | 82.48 |
| *P. phytofirmans* PsJN^T^ | *P*. *terricola* LMG20954 | 98.64 | 87.43 |
|  | *P*. *mimosarum* STM3621^T^ | 98.64 | 79.30 |
|  | *P*. *graminis* C4D1M^T^ | 98.00 | 85.44 |
|  | *P*. *megapolitana* LMG23650^T^ | 98.03 | 81.47 |
|  | *P*. *phenazinium* LMG2247^T^ | 98.26 | 82.49 |
| *P*. *terricola* LMG20954 | *P*. *graminis* C4D1M^T^ | 98.28 | 86.21 |
| *P*. *mimosarum* STM3621^T^ | *P*. *oxyphila* NBRC105797^T^ | 98.12 | 84.98 |
|  | *P*. *sacchari* LMG19450^T^ | 98.12 | 84.98 |
|  | *P*. *heleia* SA42^T^ | 98.32 | 89.91 |
|  | *P*. *silvatlantica* SRMrh-20^T^ | 98.19 | 89.16 |
| *P*. *oxyphila* NBRC105797^T^ | *P*. *ferrariae* NBRC106233^T^ | 89.05 | 84.67 |
|  | *P*. *sacchari* LMG19450^T^ | 98.93 | 89.11 |
|  | *P*. *unamae* NBRC106233^T^ | 98.16 | 85.01 |
| *P*. *ferrariae* NBRC106233^T^ | *P*. *heleia* SA42^T^ | 98.19 | 84.65 |
| *P*. *sacchari* LMG19450^T^ | *P*. *unamae* NBRC106233^T^ | 98.57 | 85.23 |
| *P*. *megapolitana* LMG23650^T^ | *P*. *phenazinium* LMG2247^T^ | 98.12 | 81.33 |
