# Supplementary material for: Volcanic Soils as Sources of Novel CO-Oxidizing Paraburkholderia and Burkholderia: Paraburkholderia hiiakae sp. nov., Paraburkholderia metrosideri sp. nov., Paraburkholderia paradisi sp. nov., Paraburkholderia peleae sp. nov., and Burkholderia alpina sp. nov. a Member of the Burkholderia cepacia Complex
Source: Front Microbiol. 2017 Feb 21;8:207. doi: 10.3389/fmicb.2017.00207 (PMC5318905; doi:10.3389/fmicb.2017.00207)
Supplement: Supplementary file 2 [file Table2.DOCX]

Supplementary Table 2. Observed Biolog GN-2 reactions for the following isolates: 1, PP521^T^; 2, *Pb*. *mimosarum*; 3, I2^T^; 4, *Pb*. *nodosa*; 5, DNBP61^T^; 6, *Pb*. *bryophila*; 7, PO-041738^T^; 8, WA^T^; w = weakly positive; v = variable.

| **Substrate**  I-erythritol  D-melibiose  Acetic acid  p-Hydroxyphenylacetic acid  Bromosuccinic acid  l-Histidine  Uroncanic acid  α-Cyclodextrin  D-Fructose  β-Methyl-D-glucoside  cis-Aconitic acid  Itaconic acid  Succinamic acid  Hydroxy-L-Proline  Inosine  Dextrin  L-fucose  D-Psicose  Citric acid  α-keto-Butyric acid  Glucuronimide  L-Leucine  Uridine  Glycogen  D-glactose  D-raffinose  Formic acid  α-keto-Glutaric acid  L-Alaninamide  L-Ornithine  Thymidine  Tween 40  Gentiobiose  L-Rhamanose  D-Galactonic acid lactone  α-keto-Valeric acid  D-Alanine  L-Phenylalanine  Phenyethylamine  Tween 80  α-D-glucose  D-Sorbitol  D-Galacturonic acid  D,L-lactic acid  L-Alanine  L-Proline  Putrescine  N-acetyl-D-Galactosamine  Myo-inositol  Sucrose  D-Gluconic acid  Malonic acid  L-Alanyl glycine  L-Pyroglutamic acid  2-Aminoethanol  N-Acetyl-D-glucosamine  α-D-lactose  D-Trehalose  D-Glucosaminic acid  Propionic acid  L-Asparagine  D-Serine  2,3-Butanediol  Adonitol  Lactulose  Turanose  D-Glucuronic acid  Quinic acid  L-Aspartic acid  L -Serine  Glycerol  L-Arabinose  Maltose  Xylitol  α -Hydroxybutyric acid  D-Saccharic acid  L-Glutamic acid  L-Threonine  D,L-α-Glycerol phosphate  D-Arabitol  D-Mannitol  Pyruvic acid methyl ester  β−Hydroxybutyric acid  Sebacic acid  Glycyl-L-aspartic acid  D,L-Carnitine  α -D-Glucose-1-phosphate  D-cellobiose  D-Mannose  Succinic acid mono methyl ester  γ−Hydroxybutyric acid  Succinic acid  Glycyl-L-Glutamic acid  γ-Amino butyric acid  D-glucose-6-phosphate | **1** | **2** | **3** | **4** | **5** | **6** | **7** | **8** |
| --- | --- | --- | --- | --- | --- | --- | --- | --- |
|  | - | + | - | + | - | - | - | - |
|  | - | - | - | - | + | w | - | + |
|  | + | + | + | + | w | - | + | - |
|  | + | + | + | + | + | + | - | + |
|  | + | + | + | + | + | + | - | + |
|  | + | + | + | + | - | - | + | - |
|  | + | + | + | + | + | + | - | + |
|  | - | - | - | - | + | + | - | + |
|  | + | + | + | + | + | + | + | + |
|  | - | - | - | - | + | + | - | + |
|  | w | + | w | v | + | - | - | + |
|  | - | - | - | - | - | - | + | - |
|  | w | v | - | + | + | + | + | + |
|  | w | + | + | v | + | + | - | + |
|  | - | - | - | - | + | + | - | + |
|  | - | + | - | - | - | - | + | - |
|  | + | + | + | + | + | + | + | + |
|  | + | v | - | + | + | + | - | - |
|  | w | + | + | + | - | - | - | - |
|  | w | + | - | v | - | - | - | - |
|  | - | - | - | - | - | - | - | - |
|  | w | + | + | + | + | + | - | + |
|  | - | - | - | - | + | + | - | + |
|  | - | + | - | + | - | - | - | - |
|  | + | + | + | + | - | - | - | - |
|  | - | - | - | - | + | w | - | w |
|  | + | + | + | + | - | - | - | - |
|  | w | v | - | + | + | + | - | w |
|  | w | v | w | + | + | + | + | + |
|  | w | v | + | - | - | - | - | - |
|  | - | - | - | - | + | - | - | + |
|  | + | + | + | + | - | - | + | - |
|  | - | - | - | - | - | + | - | - |
|  | + | - | + | + | - | + | - | + |
|  | - | v | - | + | + | + | - | + |
|  | - | - | + | - | + | + | - | + |
|  | + | + | + | + | - | + | - | + |
|  | + | + | + | + | - | + | - | + |
|  | - | v | - | v | + | + | - | + |
|  | + | + | + | + | + | w | + | - |
|  | + | + | + | + | + | + | - | + |
|  | + | + | + | + | + | w | - | + |
|  | + | + | + | + | + | + | - | + |
|  | + | + | + | + | + | + | - | - |
|  | + | + | + | + | - | w | - | + |
|  | + | + | + | + | + | + | - | + |
|  | - | v | - | v | + | - | - | + |
|  | - | - | - | - | + | + | - | w |
|  | + | + | + | + | - | - | - | - |
|  | - | - | - | - | + | w | - | w |
|  | + | v | + | + | w | w | - | + |
|  | - | v | - | - | w | - | - | w |
|  | + | v | + | + | + | + | - | + |
|  | + | + | + | + | w | - | - | - |
|  | + | v | + | - | + | + | - | + |
|  | + | + | + | + | + | + | + | + |
|  | - | - | - | - | + | + | - | - |
|  | w | - | + | + | - | w | - | + |
|  | + | + | + | + | + | + | - | + |
|  | + | + | + | v | + | + | - | w |
|  | + | v | + | + | + | w | - | w |
|  | - | v | + | + | + | w | - | - |
|  | - | - | - | - | + | w | - | + |
|  | + | - | + | + | + | + | - | + |
|  | - | + | - | - | + | + | - | + |
|  | - | - | - | - | + | + | - | + |
|  | - | - | - | - | + | + | - | + |
|  | + | + | + | + | + | + | - | + |
|  | + | + | + | + | + | + | - | + |
|  | + | + | + | v | w | - | - | - |
|  | + | v | + | v | + | + | - | + |
|  | + | + | + | + | + | + | + | + |
|  | - | + | - | - | + | w | - | + |
|  | - | - | - | + | + | w | - | + |
|  | + | + | - | - | + | w | + | + |
|  | + | + | + | + | + | + | - | + |
|  | + | + | + | + | + | + | - | + |
|  | w | + | + | v | w | + | - | + |
|  | w | - | - | - | + | - | - | + |
|  | + | + | + | + | + | + | + | + |
|  | + | + | + | + | + | + | + | + |
|  | + | + | + | + | w | - | + | + |
|  | + | + | + | + | + | + | + | + |
|  | + | + | + | + | + | - | - | + |
|  | - | v | - | + | - | + | - | - |
|  | - | v | + | v | w | w | - | - |
|  | - | - | - | - | - | w | - | - |
|  | + | - | + | + | - | - | + | - |
|  | + | + | + | + | - | - | + | + |
|  | + | + | + | v | + | + | - | + |
|  | - | - | - | - | w | - | - | + |
|  | + | + | + | + | + | + | - | + |
|  | w | v | + | + | + | w | - | + |
|  | + | + | + | + | + | - | - | - |
|  | + | - | - | v | + | + | + | + |
