# Supplementary material for: Volcanic Soils as Sources of Novel CO-Oxidizing Paraburkholderia and Burkholderia: Paraburkholderia hiiakae sp. nov., Paraburkholderia metrosideri sp. nov., Paraburkholderia paradisi sp. nov., Paraburkholderia peleae sp. nov., and Burkholderia alpina sp. nov. a Member of the Burkholderia cepacia Complex
Source: Front Microbiol. 2017 Feb 21;8:207. doi: 10.3389/fmicb.2017.00207 (PMC5318905; doi:10.3389/fmicb.2017.00207)
Supplement: Supplementary file 3 [file Table3.DOCX]

Supplementary Table 3. Substrates supporting growth in liquid culture. 1 = PP52-1^T^, 2 = I2^T^, 3 = WA^T^, 4 = DNBP6-1^T^, 5 = PO-04-17-83^T^. w = weak growth, i =

inhibitory.

| Substrate | 1 | 2 | 3 | 4 | 5 |
| --- | --- | --- | --- | --- | --- |
| Acetate | + | - | w | w | - |
| Acetone | - | - | - | - | - |
| Alanine | + | + | + | + | + |
| Arabinose | + | + | - | + | + |
| Aspartate | + | + | - | - | i |
| Benzoate | - | + | + | + | i |
| Betaine | + | - | i | + | i |
| Citrate | - | + | + | - | i |
| Dimethylamine | - | w | + | - | - |
| Formic Acid | - | + | w | - | - |
| Fructose | + | w | + | + | w |
| Galactose | + | w | + | + | + |
| Gluconic Acid | + | + | + | + | - |
| Glucuronic Acid | - | - | w | + | - |
| Glucose | + | + | + | + | + |
| Glutamate | - | - | + | w | + |
| Glycerol | + | + | + | + | w |
| Glycine | - | - | - | - | i |
| Glutarate | + | + | + | + | - |
| Hydroxybutyrate | + | w | + | + | + |
| Isopropanol | - | - | - | - | - |
| Lactate | + | + | + | + | + |
| Lactose | - | - | - | + | - |
| Malic Acid | + | + | w | + | w |
| Malonic Acid | + | w | w | w | - |
| Maltose | - | - | - | w | - |
| Mannitol | + | + | + | + | + |
| Mannose | + | w | + | + | + |
| Methanol | - | - | - | - | - |
| Methylamine | - | - | - | - | - |
| Phenlyalanine | - | + | - | - | i |
| Phthalate | - | - | i | i | i |
| Proline | + | + | + | + | w |
| Propionate | + | + | w | + | i |
| Ribose | + | + | - | + | + |
| Succinate | + | + | + | + | - |
| Tartrate | + | + | w | + | + |
| Trimethylamine | - | w | + | - | - |
| Valine | - | - | + | - | - |
