# Supplementary figures and images for: Volcanic Soils as Sources of Novel CO-Oxidizing Paraburkholderia and Burkholderia: Paraburkholderia hiiakae sp. nov., Paraburkholderia metrosideri sp. nov., Paraburkholderia paradisi sp. nov., Paraburkholderia peleae sp. nov., and Burkholderia alpina sp. nov. a Member of the Burkholderia cepacia Complex
Source: Front Microbiol. 2017 Feb 21;8:207. doi: 10.3389/fmicb.2017.00207 (PMC5318905; doi:10.3389/fmicb.2017.00207)

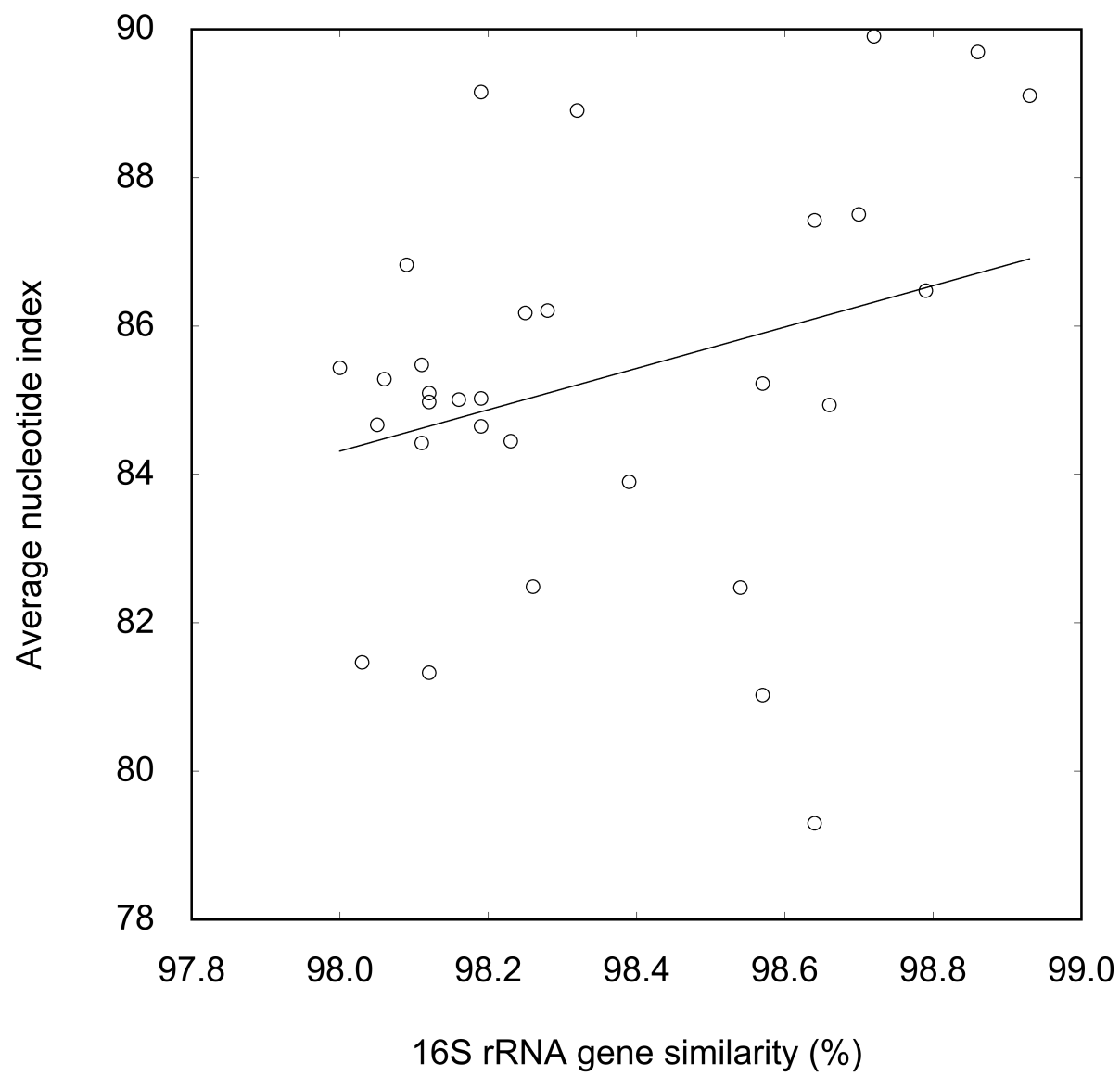

Supplement: Supplementary file 4 [file Image1.PDF]
